# Fluoro-edenitic fibres in the sputum of subjects from Biancavilla (Sicily): a pilot study

**DOI:** 10.1186/1476-069X-5-20

**Published:** 2006-06-16

**Authors:** Maria Grazia Putzu, Caterina Bruno, Amerigo Zona, Marilena Massiccio, Roberto Pasetto, Pier Giorgio Piolatto, Pietro Comba

**Affiliations:** 1 Department of Traumatology, Orthopaedics and Occupational Medicine – University of Turin, Via Zuretti 29 - I-10126 Turin, Italy; 2 Department of Environment and Primary Prevention – Istituto Superiore di Sanità, Viale Regina Elena 299 - I-00161 Rome, Italy

## Abstract

**Background:**

An excess of mortality for malignant neoplasms of the pleura in Biancavilla, promoted an investigation for pleural mesothelioma, disclosing 17 cases. As the absence of known sources of asbestos exposure, a local stone quarry, located near the inhabited area, used for the extraction of building materials, was investigated. Amphibolic fibres were found in the quarry and identified as fluoro-edenite "new end-member of the edenite / fluoro-edenite series" and recognized as the fluoro-edenite holotype by International Mineralogical Association – Commission on New Minerals and Mineral Names. A pilot study was performed to verify the feasibility of using spontaneous sputum as an exposure indicator for these fibres, in a context in which the use of aerosol-induced sputum technique would not be easily accepted.

**Methods:**

Hypothesizing a behaviour of the new fibre analogous to that of asbestos, the determination of the free fibres and the ferruginous bodies in spontaneous sputum was carried out. Phase Contrast Optical Microscope and an Environmental Scanning Electron Microscope fitted with X-ray energy dispersive analysis system (micro-analysis) were used to examine the samples. The criteria for inclusion in the study were: 1) subjects hospitalized for exacerbation of chronic obstructive pulmonary disease symptoms, 2) age ≥ 45 years, 3) residence in Biancavilla for at least 30 years.

**Results:**

The preliminary findings are related to 12 subjects (7 females and 5 males). Uncoated fibres (with length > 5 μm, diameter < 3 μm, aspect ratio 3.1) and ferruginous bodies were searched. Six out of twelve subjects (4 females, 2 males) had at least one of the three samples positive for the presence of fluoro-edenite, confirmed by micro-analysis. The fibre concentration found in the sputum ranged from 0.04 to 10 fibres/g; the length from 20 to 40 μm, the diameter was < 0.5 μm.

No ferruginous bodies were found in any of the samples. The four women with a positive sample were housewives. Of the two men with a positive sample, one was a farmer and the other a mason. Therefore, it may be assumed that the exposure to fluoro-edenitic fibres was mainly environmental.

**Conclusion:**

The occurrence of the pleural mesothelioma cases and the presence of fluoro-edenitic fibres in spontaneous sputum, evidence the need to study the biological activity of fluoro-edenitic fibres and the implementation of epidemiological monitoring systems.

## Background

An excess of mortality for malignant neoplasms of the pleura during the period 1988 to 1992 in Biancavilla, a rural town located on the southwest side of the Etna volcano [1], promoted an investigation disclosing 17 ascertained cases of pleural mesothelioma until the end of 1997 [2]. In the light of the absence of known sources of asbestos exposure, a local stone quarry, located near the inhabited area, used for the extraction of building materials, was investigated. Amphibolic fibres were found in the quarry and identified as fluoro-edenite "new end-member of the edenite/fluoro-edenite series" and recognized as the fluoro-edenite holotype by International Mineralogical Association – Commission on New Minerals and Mineral Names (IMA-CNMMN) [3]. Fluoro-edenite was thus considered to be the causal agent of the observed mesothelioma cluster [4]. Fluoro-edenitic fibres were found in the autopsy samples of lung tissues of an 86-year-old woman who died of mesothelioma [2], in sheep lung specimens [5], and in sheep lymph nodes [6]. Further investigations were performed to evaluate chemical and physical characteristics of the new mineral [7], and to examine geo-volcanological, mineralogical and environmental factors of quarry materials [8].

The termination of quarrying activity and the asphalting of roads previously paved with local soil materials were among the public health recommendations adopted to decrease the exposure to the fluoro-edenite, and were implemented in 2003.

Studies on the effects fluoro-edenite might have on human health were undertaken. An ecological study performed to investigate the association between mortality from chronic obstructive pulmonary disease (COPD) and exposure to fluoro-edenite in thirty-six municipalities located in the volcanic area of Mount Etna, found a significant association between COPD mortality and pleural neoplasm mortality among women, suggesting an etiologic role of fluoro-edenite in nonmalignant respiratory diseases [9]. A recent study confirmed the increase of the incidence of pleural mesothelioma in the period 1998–2004 [10].

The biological behaviour of the new mineral, in vivo and in vitro, is being studied by various research groups [11,12].

In order to investigate fluoro-edenite respirability and persistence, a study was planned by the Department of Occupational Medicine of the University of Turin, the Italian National Institute of Public Health, the Local Health District and the Biancavilla Hospital. Hypothesizing a behaviour of the new fibre analogous to that of asbestos and other mineral fibres in the environment [13,14], it was decided to carry out the determination of the free fibres and the ferruginous bodies in sputum. To verify the feasibility of using spontaneous sputum as an exposure indicator for these fibres, a pilot study was performed. Sputum examination was preferred to the bronchial alveolar lavage (BAL) technique, as it is non-invasive. Moreover the method has been validated in subjects exposed to asbestos in the workplace [15].

## Methods

Since the sensitivity of sputum examination for fibres and ferruginous bodies as a marker of asbestos exposure is low, even in occupationally exposed people [16], the criteria for inclusion in the study were:

1) subjects hospitalized for exacerbation of COPD symptoms, to collect the highest possible quantity of sputum,

2) age ≥ 45 years,

3) residence in Biancavilla for at least 30 years.

Three spontaneous sputum samples from the morning cough, collected over 3 days not necessarily consecutive, were taken for each subject, following a methodology already used [17,18]. The containers were weighed before analysing the sample, to weigh the sputum and determine the sample validity. The sample was considered valid, i.e. not polluted with oropharingeal debris or saliva, when containing less than 20% of squamous cells, more than 5–10 neutrophil cells for microscopy field and more than 50% vital cells. After examination under an inverted microscope, a part of the sample was placed into a glass test tube. It was weighed, and dithiothreitol diluted in 1:10 distilled water then added according to the manufacturer's instructions, in an equal volume (in ml), to double the weight of the sputum portion (in mg). The test tube was placed in an oven at 37°C for 20 minutes and shaken every 5 minutes. After repeated pipetting, it was further diluted with phosphate buffered saline (PBS) to a volume equal to the sputum plus dithiothreitol. The cells suspension was filtered through a 52 μm nylon gauze to remove debris and mucus and the vitality quantified (exclusion Trypan Blue method). The material was centrifuged at 1000 RPM × 10 minutes. The surnatant was frozen at -70°C, the pellet placed in PBS as before, and the total cell count measured using a hemocytomer. If the sample was considered valid, the surnatant was digested in 2 ml of hydrogen peroxide at 30% and 2 ml of sodium hypochlorite for 24 hours to eliminate all the organic components, to examine it for mineral fibres and ferruginous bodies.

After digestion, the surnatant was filtered through a Millipore^® ^membrane filter (mixed esters of cellulose, 25 mm diameter, 0.45 μm pore filter), which was washed in water and then pre-heated at 60°C.

Two membranes were prepared for each sample: one, made transparent with acetone vapours and examined under a Phase Contrast Optical Microscope (PCOM) at magnifications of 450×, the other, mounted on a stub, was examined under an Environmental Scanning Electron Microscope (ESEM) fitted with X-ray energy dispersive analysis system (XL 30 Philips with EDX Digital Controller). Magnifications ranged from 1,000 to 8,000×. The samples were uncoated because the ESEM allows the examination of specimens in the presence of gases and so the specimens can be viewed without preparation. EDX automatically removes background component for each element, at every point in a map, increasing contrast and making easier to find areas where the element of interest may be present in lower concentrations.

## Results

The preliminary findings are related to 12 subjects (7 females and 5 males). All samples were acceptable, according to the above defined criteria. Uncoated fibres (with length > 5 μm, diameter <3 μm, aspect ratio 3.1) and ferruginous bodies were searched.

**Figure 1:**
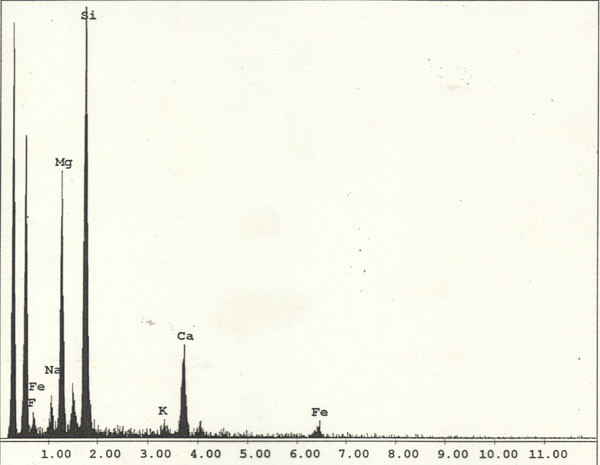
EDAX spectrum – Fluoro-edenite.

**Figure 2:**
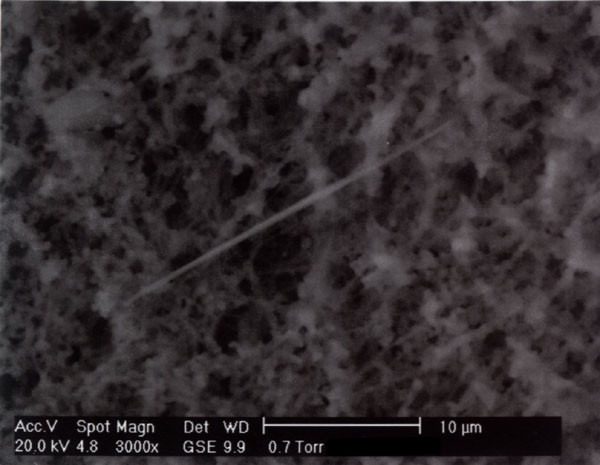
Fluoro-edenitic fibre.

The fibres observed belonged to two different mineralogical types: some had an EDAX spectrum containing F, Si, Ca, Fe (figure 1) (compatible with fluoro-edenite, figure 2), others had an EDAX spectrum containing P and Ca (compatible with apatite).

Six out of 12 subjects had at least one of the three samples positive for the presence of fluoro-edenite, confirmed by micro-analysis. The fibre concentration found in the sputum ranged from 0.05 to 10 fibres/g (table 1). Two out of six subjects positive for the presence of fluoro-edenitic fibres were also positive for the presence of apatite fibres (concentration found in the sputum ranges from 0.1 to 1.7 fibres/g, table 1). The length of fluoro-edenitic fibres ranged from 20 to 40 μm, the diameter was <0.5 μm. The length of apatite fibres ranged from 10 μm to 20 μm, the diameter from 0.6 μm to 1.5 μm. No bundles of fibres for either of the fibre types, but only single fibres were found.

**Table 1:** Results of sputum analysis

**Age**	**Sex**	**R**	**Main work**	**Smoke**	**N**	**P**	**Ap**	**F**
81	M	81	farmer	Yes	3			
74	M	74	farmer	Yes	1	2		1.6 0.5
69	M	69	farmer	Yes	3			
65	M	65	farmer	-	3			
73	M	34	builder	Yes	1	2		0.04 0.06
84	F	84	housewife	-	1	2		1 10
75	F	75	housewife	-	3			
75	F	75	housewife	-	1	2	1.7	0.07
67	F	67	housewife	-	2	1		0.7
64	F	64	housewife	-		3	0.5	1
56	F	56	housewife	-	3			-
43	F	43	housewife	-	3			-

R Residence (yrs)

N Number of negative samples

P Number of positive samples

Ap Apatite fibres in sputum (fibre/g)

F Fluoroedenitic fibres in sputum (fibre/g) (Three samples for each subject)

No ferruginous bodies were found in any of the samples. The four women with a positive sample were housewives. Of the two men with a positive sample, one was a farmer and the other a mason. Therefore, it may be assumed that the exposure to fluoro-edenitic fibres was mainly environmental.

## Discussion

Notwithstanding some Authors showed that aerosol-induced sputum technique was more effective for detecting ferruginous bodies than spontaneous sputum specimens [19], we decided to test the latter one method because it was more acceptable, considering the local social context. Despite the small group studied, these results prompt us to continue the study, for several considerations. The presence in the sputum of either asbestos or other natural mineral fibres confirms recent and past exposure [15], whereas the absence of ferruginous bodies cannot rule out the asbestos exposure. The ferruginous bodies (fibre / particle coated with both protein and iron) are considered to be the result of a protective mechanism of the host to reduce the fibre toxicity. Only a fraction of fibres reaching the alveoli becomes coated forming ferruginous bodies and, in case of amphibole minerals, fibres shorter than 10 μm rarely become coated [20].

Even though the studied group had had a long-term environmental exposure, and, therefore, the formation of ferruginous bodies could have been hypothesized, however, all the samples were negative. This finding may be due to either the small number of subjects under study, or to the properties of the fibre itself, which might not be capable of inducing the mechanism of ferruginous body formation. In a study undertaken to evaluate the usefulness of uncoated asbestos fibre in sputum to distinguish occupational exposed groups from the general population, uncoated fibres were found in 10 out of 12 former amosite asbestos workers and in only one out of 12 age-matched controls [21].

## Conclusion

The observation of the presence of fluoro-edenitic fibres in the sputum of 6 out of 12 COPD patients examined in Biancavilla points to a relatively important environmental exposure; in this frame further action aimed at environmental reclamation, continuing exposure assessment, and evaluation of the suggested relation between fluoro-edenitic fibres and COPD are warranted. Furthermore, it could be interesting to investigate the presence of interstitial lung fibrosis in this population; cytopathological evaluation of sputum samples would be performed as well.

## Abbreviations

BAL – Bronchial alveolar lavage

COPD – Chronic obstructive pulmonary disease

ESEM – Environmental Scanning Electron Microscope

IMA-CNMMN – International Mineralogical Association – Commission on New Minerals and Mineral Names

PBS – Phosphate buffered saline

PCOM – Phase Contrast Optical Microscope

## Competing interests

The authors declare that they have no competing interests.

## Authors' contributions

MGP: design and implementation of monitoring of fibres in sputum.

CB: field study: selection of study subjects, organization of samples collection, evaluation of findings in light of available scientific evidence

AZ: evaluation of medical aspects of study subjects

MM: execution of fibre measurements in sputum samples

RP: epidemiologic expertise

PGP: scientific supervision

PC: expertise on fluoro-edenite epidemiology and toxicology

All authors read and approved the final manuscript.
